# The data obtained during the analysis of clinical blood samples for children acute lymphoblastic leukemia patients with severe side-effects

**DOI:** 10.1016/j.dib.2017.03.016

**Published:** 2017-03-12

**Authors:** Andrey Zyubin, Anastasiya Lavrova, Maksim Demin, Alina Pankina, Svetlana Babak

**Affiliations:** a Immanuel Kant Baltic Federal University, A. Nevskogo St. 14, Kaliningrad 236016, Russia; b Research Institute of Phthisiopulmonology, Polytechnicheskaya 32, Saint-Petersburg 194064, Russia

**Keywords:** Child acute lymphoblastic leukemia, Pathological processes, Bio-markers, Spectral data (MALDI-TOF)

## Abstract

The present work contains data obtained during the analysis of blood samples obtained from patients (number: 102, age: 0–2 years old) with confirmed acute lymphoblastic leukemia diagnosis. Among these total number, 48 patients who received a chemotherapy including 9 patients with side-effects were chosen. Final data include a table of protein masses (biomarkers of pathological processes at the chemotherapy) obtained by the MALDI-TOF spectroscopy from blood of 9 chosen patients (0–2 years old).

**Specifications Table**

**Table:** 

Subject area	*Bio-medicine*
More specific subject area	*Identification of the blood proteins by the methods of MALDI-TOF mass spectroscopy*
Type of data	*2 Tables, Text file*
How data was acquired	*PROTEAN IEF Сell (Bio-Rad, USA),*
	*Сentrifuge Eppendorf 570 (Eppendorf, Germany)*
	*Autoflex Speed MALDI-TOF/TOF (Bruker, USA)*
Data format	*Analyzed*
Experimental factors	Clinical and biochemical analysis was conducted to choose the blood samples with side-effects of chemotherapy. Isolated membrane, mitochondrial, nuclear and subcellular fractions have been obtained by the methods of sequential lysis and centrifugation. The total protein was obtained from each fraction, and then proteins were separated and placed to the gel.
Experimental features	Proteins in gel were isolated by the lysis. Identification of proteins and determination of its masses were conducted by the method of mass-spectrometry.
Data source location	Kaliningrad, Russian Federation
Data accessibility	Data are presented with this article

**Value of the data**

• Data could be useful for physicians working in the field of personalized medicine.
• These data could be considered as “first step” toward further experiments analyzing more proteins or subcellular fractions at the treatment of ALL among small children and comparing with data obtained in other labs.
• Using these data, it will be possible to join efforts to create a unified database of biomarkers of pathological processes at the medical cure of children ALL.
• Data, obtained for 9 patients with severe side effects could be useful for therapy of ALL, since identified markers could be used for development of recommendations for individual patient treatment.

## 1 Data

The presented data include information on the proteins of different subcellular fractions in blood and their concentration (Table 1), and proteins masses obtained by the mass-spectroscopy (Table 2).

## 2 Experimental design, materials and methods

The experiment׳s planning, design and data processing correspond to the protocols given in Refs. [1], [2].

### 2.1 Samples collection

The data were taken from plasma proteins of 102 patients (69 boys and 33 girls) with the verified diagnosis of acute lymphoblastic leukemia. All patients were under pharmacotherapy and their adult representatives gave informed consent for inclusion in the data processing and reporting.

The average age of all patients was 2 years and four months, of which the average age of girls at the beginning of the analysis was (2.5±0.65) years, boys - (2.4±0.6) years. The group of boys and girls was subdivided accordingly to the indicator of body surface area (BSA): BSA smaller than physiological standard and standard BSA. For girls only, four patients had the age-related physiological norm of BSA (0.57±0.06) cm^2^; BSA of 27 patients was (0.44±0.03) cm^2^, 15 boys corresponded to the physiological norm of BSA equal to (0.54±0.03) cm^2^, and the remaining 54 boys fallen into the group (0.45±0.03) cm^2^. In this case, the high risk of the relapse was observed in 30% of cases (*n*=32), including 12% for girls (4 people) and 88% (*n*=28) for boys, respectively. Patients with BSA under the physiological norm dominated among this sample; in particular, only one of 4 girls had a BSA standard, and 8 boys of the whole studied group (28 persons).

Clinical and biochemical analysis of all samples was conducted to choose patients with side-effects. The most severe damage of liver and kidney functions was observed in 9 patients (1 female and 8 males) with an initial deficit of BSA. A blood test was re-evaluated after the start of chemotherapy course (14–15 h) at the growth rates of aminotransferase and creatinine levels and the break of electrolyte composition of blood and metabolic processes.

### 2.2 Isolation of mononuclear cells

To separate mononuclear cells from blood samples, vacuum tubes of the type K-EDTA (including EDTA and K2-K3-EDTA and BD) were used. The tubes were filled with blood up to the level indicated on the factory label by hairline. After blood addition into the vacuum tubes, each of them was immediately turned over 8 times, and placed in a tube-rack. Samples were stored at room temperature not more than 3 h before experiment.

### 2.3 Isolation of serum

The samples were stored before processing at room temperature. The processing of blood clot has been started not earlier than 30 min after the blood sampling, and not later than 2.5 h after it. Next, the centrifugation (Сentrifuge Eppendorf 570) was performed at the speed of 2000 rev/min for 10 min at room temperature. The obtained serum (1 ml) was collected in cryovials, which had the volume of 1.8–2.0 ml. The cryovials were stored at −80 °C.

Overall, 48 clinical samples of peripheral blood were obtained from patients undergoing treatment with ALL diagnosis using the algorithm of blood samples profiling described above. Protein concentration in the extracts was determined by the method of Bradford [1]. 2D gel electrophoresis was performed according to the standard protocol [2].

Blood mononuclear cells were isolated from the obtained samples by the fractionation in Ficoll/Urografin density gradients (1.077 g/cm^3^). Membrane, mitochondrial, nuclear, cytoplasmic subcellular fractions were separated using the methods of sequential lysis and centrifugation; proteins were extracted from each subcellular fraction.

To define proteins concentration with the spectrophotometer Shimadzu UV-3600 (Japan), the Bradford solution was prepared: 100 mg of Coomassie G-250 were dissolved in 50 ml of ethanol, and subsequently 100 ml of phosphoric acid and 600 ml of water were added; the resulted mixture was filtered through a paper filter. The purity of the Bradford solution was determined visually by the presence of characteristic brown color, which becomes blue with the presence of protein. The volume of each sample was adjusted to 0.5 ml of water, 0.5 ml of reagent, stirred, and left at room temperature for no longer than 25 min, until the blue color emergence; the prepared sample was placed in 1 ml cuvette for the measuring OD_595_.

### 2.4 Determination of protein concentration

The protein concentration was calculated in the subcellular fractions of patients׳ blood using a calibration curve constructed from BSA (bovine serum albumin). The range of BSA concentrations was from 0.1 mg/ml to 10 mg/ml. Results are presented in Table 1.

Proteins were isoelectric focused on immobilized pH gradients then separated by the vertical gel-gradient electrophoresis after addition of sodium dodecyl sulfate. Gels were fixed with 40% ethanol, and the proteins were visualized by adding silver to the gels in acidic solution. The gel was soaked for 30 min in a 10% aqueous solution of glutaraldehyde, washed for 10 min in 0.5–1 l of water (with two changes) and left to swell for at least 2 h in the water. Then, the water was decanted and a freshly prepared solution of ammonia silver was added to the gel. To get 100 ml of this solution, 14.ml fresh ammonia solution was added to 0.36 ml of 21% NaOH solution with further slower addition of 4 ml of 19.4% solution of silver nitrate (20 g AgNO_3_ + 100 ml of water) at constant shaking. Thus, a temporarily brown precipitate was formed; after its dissolution, water was added to get the solution volume of 100 ml. To create a good and regular color and to avoid sticking of the gel to the bottom, the "free floating" conditions for the gel were achieved in the bath with the size of 20×20 cm^2^ poured by 150 ml of ammonia solution of silver. The shaking speed was increased up to 75 rev/min at this time. The staining time did not exceed 15 min in order to prevent the pattern formation on the gel surface.

The gel was removed from the solution and washed for 2 min; to avoid a gel adhesion to cuvette, the gel was transferred with water. Then, the gel was transferred to a freshly prepared solution containing 0.005% citric acid, and 0.019% formaldehyde (prepared by diluting the commercially available 3׳8% formaldehyde in 10% methanol); and wherein the bands of proteins were appeared. The gel was removed, when the background became dark, and washed with water for an hour with three changes at the constant stirring. The gel background was brightened by the pre-fixation of proteins in 10% acetic acid with 50% methanol for 30 min followed by washing of the gel in 7% acetic acid, with 5% methanol during one night. The gels were frozen for the consequent isolation of protein spots.

### 2.5 Description of the MALDI-TOF experiment

Identification of proteins was performed by mass spectrometry (Autoflex Speed MALDI-TOF/TOF (Bruker, USA)) determining tryptic peptides released during the lysing of protein spots isolated from the gel. Trypsinization was held in the lyophilized gel fragments with the consequent elution and purification of peptides located on microcolumns filled with the surface-modified silica gel. The gel pieces were washed twice for thirty minutes at 37 ° C in 100 μl of 40% acetonitrile dissolved with 0.1 M of ammonium bicarbonate. The solution and surplus of liquid were removed, and then sample was dehydrated with 100 μl of acetonitrile. Next, acetonitrile was blended, and the sample was dried at room temperature. As a next step, the modified trypsin solution of 15 μg/ml concentration dissolved in 0.05 M NH_4_HCO_3_ was prepared; 3 μl of the modified trypsin solution was added into the sample. The sample was placed into an incubator and had been incubated at 37 °C for 12 h.

The solution of 0.5% tetra fluoride (TFA) in an aqueous solution of acetonitrile was prepared. The working solution was prepared in 20% aqueous acetonitrile with 0.5% trifluoroacetic acid from 20 mg/ml of 2,5-dihydrobenzoic acid. After 12 h, 7 l of 0.5% TFA solution were added to the sample taken out fro the incubator. This solution (2 μl) was mixed with 0.3 μl of 2.5-dihydrobenzoic acid and the obtained mixture (2 μl) was added to a target. The sample was dried at room temperature. The target was irradiated with a high-frequency laser.

Measurements were performed in the positive ion mode. The settings of the scientific unit were: reflectron in the range from 700 to 4500 V; common dispersing potential of reflectron mode up to 25 kV. The accelerating potentials of parent ion for mass spectrometry in the tandem mode and of fragment ion were 47 + 7 kV and 28 kV, respectively. Quality control check was performed by the trypsin autolysis peaks determination. Accuracy of the mass measurements was in the range of ±2 Da. Control data for healthy patients was not performed in case of negative markers expression [3]. Mass spectra were processed using Treatment Flex Analysis 2.0 program. Identification of proteins was carried out using PDB (protein data bank) and Matrix Science (Mascot) database, see Table 2.

## Figures and Tables

**Table 1:** Total protein concentration in the subcellular fractions of the blood of patients with different side-effects.

Subcellular fractions	Severe side-effects (*n*=9)	Light side-effects (*n*=48)
Membrane	0,5+0,07 ng/ml	0,3+0,02 ng/ml
Mitochondrial	0,2 +0,05 ng/ml	0,11+0,09 ng/ml
Nuclear	0,07+0,02 ng/ml	0,07+0,005 ng/ml
Cytoplasmic	0,9+0,113 ng/ml	0,9+0,098 ng/ml

**Table 2:** Protein identification and analysis based on experimental data, PDB and Mascot search (Matrix Science).

Molecular mass (2D) KDa	Protein name (Mascot search, PDB)	Protein mass, Da (Mascot search, PDB)	Protein isoelectric point, PH	Protein score (Mascot search)
26	ARL14_HUMAN,	Mass: 21608	8,7	39
13	THIO_HUMAN, Mass:	Mass: 11730	4.7	69
	11730 4.7	Mass: 12574	7.0	
	HV303_HUMAN, Mass: 7.0			30
22	SOD2_HUMAN, Mass: 8.3	Mass: 24707	8.3	73
	PCLI1_HUMAN Mass: 5.8	Mass: 28253	5.8	26
20	PRDX2_HUMAN Mass: 5.7	Mass: 21878	5.7	172
	N2DL3_HUMAN Mass: 8.0	Mass: 27931	8.0	37
	KCY_HUMAN Mass: 7.9	Mass: 22208	7.9	36
	THAP1_HUMAN Mass: 8.2	Mass: 24928	8.2	26
	CWC15_HUMAN Mass: 5.5	Mass: 26608	5.5	32
28	PGAM4_HUMAN Mass: 6.5	Mass: 28759	6.5	33
	PGAM1_HUMAN Mass: 7.0	Mass: 28786	7.0	43
	CISH_HUMAN Mass: 6.2	Mass: 28645	6.2	25
	PAB1L_HUMAN Mass: 8.6	Mass: 29857	8.6	29
